# P-1279. Understanding Vaccine Hesitancy and Health Literacy among Parents in the United States

**DOI:** 10.1093/ofid/ofae631.1460

**Published:** 2025-01-29

**Authors:** Amanda Eiden, Dong Wang, Yi Zheng, Paula Frew

**Affiliations:** Merck, Philadelphia, PA; Merck & Co., Inc., San Diego, California; Merck & Co., Inc., San Diego, California; Merck & Co., Inc., San Diego, California

## Abstract

**Background:**

Vaccination effectively prevents diseases and reduces the health system burden. However, vaccine hesitancy persists and affects vaccination uptake. We aim to better understand the level of vaccine hesitancy and its associated factors among parents.

**Methods:**

A cross-sectional survey was conducted (January 2022) among 692 parents with children under 18 years old in the US. Demographic characteristics were described by level of vaccine hesitancy. Logistic regression models were fitted to assess the associations of vaccine hesitancy towards children’s vaccines with factors related to literacy, information seeking behavior, attitudes and beliefs, and access to care. We compared vaccine hesitancy levels across vaccine preventable diseases using Chi-Square tests. Odds ratios (OR) with their 95% confidence intervals (CI) and p-values were reported.

**Results:**

Overall, more hesitant parents were more likely to be younger, male, and Hispanic. After adjusting for age, gender, race/ethnicity, education, income, and region, parents more familiar with children’s vaccines had lower odds of being hesitant (OR= 0.82, 95% CI: 0.69 - 0.97). Open discussion with healthcare professionals, trust in vaccination authorities, belief that vaccines benefit children, and easy access to pharmacy were significantly associated with less hesitancy. Higher odds of being hesitant were observed among parents who were more cautious in decision-making or had information seeking challenges, and those who had concerns regarding vaccine safety, efficacy, associated side effects, or frequent vaccine schedules. Parents were more hesitant towards seasonal vaccines (e.g., COVID-19, influenza) compared to routine vaccines (e.g., tetanus-containing, MMR).

**Conclusion:**

This study informs public health entities and medical practitioners where to target positive vaccine-related messaging and resources to enhance parental vaccine literacy and build trust in vaccines.

**Disclosures:**

**Amanda Eiden, PhD, MBA, MPH**, Merck & Co., Inc.: Stocks/Bonds (Private Company) **Dong Wang, PhD**, Merck & Co., Inc.: Stocks/Bonds (Private Company) **Yi Zheng, PhD, MPH**, Merck & Co., Inc.: Stocks/Bonds (Private Company) **Paula Frew, PhD, MA, MPH**, Merck & Co., Inc.: Stocks/Bonds (Private Company)

